# The use of peer-teaching in general practice: advantages and lessons learned

**DOI:** 10.5116/ijme.5f28.798e

**Published:** 2020-08-21

**Authors:** Kadri Suija, Anu Sarv, Tarmo Loogus, Ruth Kalda

**Affiliations:** 1 Institute of Family Medicine and Public Health, Faculty of Medicine, University of Tartu, Estonia; 2 Faculty of Medicine, University of Tartu, Estonia

**Keywords:** Peer-teaching, general practice, lessons learned, Estonia

## To the Editor

Several studies have shown that early and frequent contact with general practice during one’s undergraduate curriculum has the potential to increase a student’s interest in primary care.^1^^-^^3^ Concurrently, higher education is increasingly utilizing novel teaching techniques to enhance learning by increasing the involvement of students in the teaching and learning process. One approach is the use of peer-assisted learning. Peer teaching (also known as peer-assisted learning, peer tutoring, and peer assessment) can be defined as an educational arrangement in which a student teaches one or more fellow students.^4^ Peer teaching influences teaching and learning in a broader way by providing an additional possibility to introduce a speciality, by organizing the course in a special setting or by involving skills important in the field.

In this paper, we share lessons learned from our experience in using peer-assisted learning in teaching clinical skills for undergraduate medical students in general practice. We hope that it will encourage other medical educators to include peer teaching into general practice teaching more often. In our case, the initiative to organize this course came from students due to a lack of practical skills training in our undergraduate teaching. Consequentially, we started a new, optional course at the Department of Family Medicine for medical students. We decided to concentrate on practical clinical skills important in general practice. Currently, this course has taken place six times. Among our students, there were individuals with professional backgrounds in physiotherapy or nursing; therefore, it was easy to include their previous professional skills in the teaching process. Three experienced clinical teachers, who were also general practitioners, were involved with the tutorship of this course. The role of the general practice teachers during the preparations was to determine the teaching aims, assess the suitability of the materials used by the peer teachers, and give practical advice and feedback to their teaching. Peer teachers did not receive payment for their teaching, but received credit points instead. The course took place for one full day and consisted of different sessions (e.g. knee examination, cannulation, minor surgery). Each session was 45 minutes with a 15-minute break between sessions. We regularly collected feedback from the participating students, and also conducted focus groups with peer teachers and general practice teachers to investigate the peer-assisted learning experience.

We were surprised how satisfied the students were with the whole course. They, especially, valued ability to practice. The need for more examples and practical illustrations were mentioned as the shortcomings, which reflects the lack of practical and teaching experience of peer teachers. On the other hand, our students were pleased with the teaching of peer teachers. They even expressed the feeling that a peer teacher is a more effective teacher than a ‘real’ teacher because the peer teacher can understand the students better and can, therefore, supervise learning more effectively. Our peer teachers found that this experience gave them a much broader picture of medical teaching and work in general practice on the whole.

We learned that peer teachers require significant support from experienced teachers (technical, knowledge, and support on the emotional level). It could be a challenge for faculty to give the right amount of support and, at the same time, have control over the process to guarantee a safe learning environment without interfering with the independence and initiative of peer teachers. This assumes sufficient preparation time and dedicated faculty members. Our general practice teachers found that peer teachers’ enthusiasm, conscientiousness, and eagerness in preparation were outstanding. Thus, we can observe that mutual trust between students and faculty increased.

We found, especially important that peer teachers reported that they learned transferable skills during this experience, such as collaboration, communication, and time management. All of these will be beneficial for them in the future when they become senior doctors, and if they need to supervise medical students or junior doctors.^5^

We conclude that peer-assisted learning is important. It enables peer teachers to improve their theoretical knowledge along with their practical skills. We learned that peer teachers require prior teacher training. Participation in a special, peer-mentor program significantly increased medical students’ confidence in their teaching skills.^6^ We also must consider how to raise the awareness of peer teachers about their own development and learning; how to make this development more apparent during the process; and, how to motivate new peer teachers. There is evidence that more teaching (courses, clerkship) in general practice may be factors in choosing general practice work in the future.^1^ Some of our peer teachers have continued their teaching activity for more than one year, which proves their interest to general practice. Based on the course’s positive evaluation and popularity among students, we think that peer-assisted teaching succeeded to strengthen the role of general practice in medical education.

### Acknowledgements

The authors would like to thank the peer teachers and students for their participation in this study.

### Conflict of Interest

The authors declare that they have no conflict of interest.
